# Pedagogical implications of pragmatic HRM research

**DOI:** 10.3389/frma.2024.1374628

**Published:** 2024-06-11

**Authors:** Dieu Hack-Polay

**Affiliations:** ^1^Department of Management, University of Lincoln, Lincoln, United Kingdom; ^2^Department of Graduate Studies, Crandall University, Moncton, NB, Canada

**Keywords:** pragmatism, HRM research, collaborative model, theory and practice, interpretivism, sociological imagination

## Abstract

This chapter examines the interplay between human resource management theory and human resource management practice. It advocates that effective human resource management practice and theory are intrinsically intertwined, and this indispensable link ought to be central to the pedagogy of management research methods. Through greater embeddedness of the institutional and societal context in research method teaching, students can develop as scholars who understand their roles as facilitators of dialogue between researchers and a significant part of their audience (practitioners). The chapter conceptualizes this perspective as a collaborative model in human resource management research, which then must hold centrality in the teaching of research methods in our university and college classrooms.

## Introduction

We can understand globalization and global HRM better with increased in-depth research in specific cultural contexts, particularly when we are being drawn into the global domain and marketplace (Brewster and Sparrow, 2007). In this regard, as Wright et al. (2015) explain, and from this study's perspective, the growing internationalization of research in international human research management (IHRM) is one of the significant developments in the last 30 years, purporting to move toward a more critical human resource management (CHRM), with increasing research emerging in many different countries, not just the West. This is starting to embed the intricate business environment and employment contexts. These are also increasingly having visibility in top level international academic and practice-based articles. The necessary CHRM perspective is further reasserted by Delbridge and Keenoy (2010), who see two possible sources for CHRM (external influence and contribution of internal critics), further reinforcing the authors' conception of pragmatism in international human research management.

While reviewing HRM developments in the past three decades, Kaufman (2015) suggests that the HRM field needs to “rebalance” itself by reducing the emphasis on “scientism” but encouraging and increasing field investigation and participant–observer methods. Such a research direction will pay more attention to the external side of HRM and integrate perspectives emanating from associated social science disciplines. He calls for broadening research from the present predominant focus on best-practice success stories to include more representative and even less attractive case studies (Kaufman, 2015) internationally. Hayton (2015) states that action research may offer that model in a more hypothetical-deductive fashion, which simultaneously examines questions of practice while engendering new theories for future testing. However, he also laments the fact that action research has not enjoyed a greater degree of success in terms of penetrating scholarly journal space, barring a few exceptions (Hayton, 2015). Cascio (2015) implies that the development of systematic (grounded) theory about the causes of problems and the means for change cannot be accomplished through narrowly focused and precise positivistic methods.

**Figure 1:**
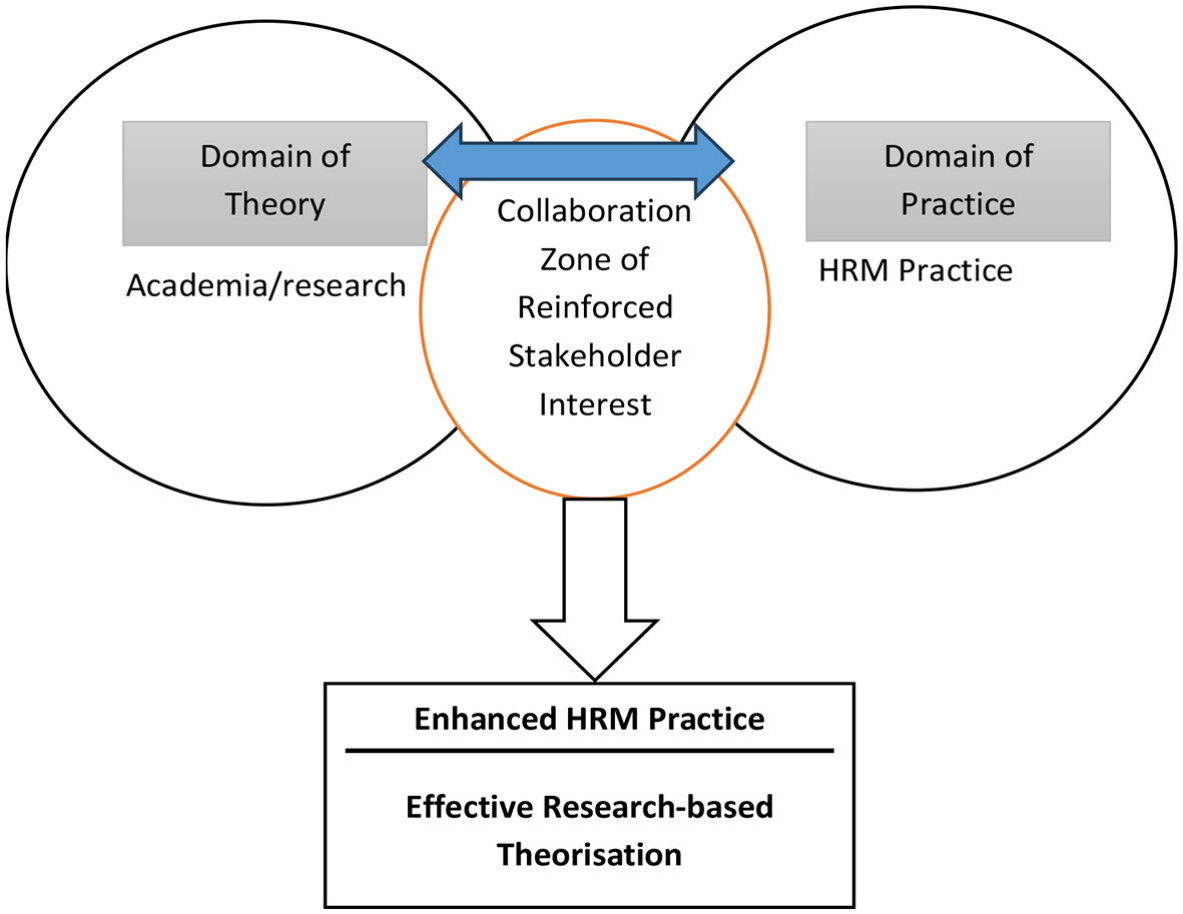
Collaborative model—Duality theory and practice in HRM.

The purpose of this chapter is to offer a framework for research into international human resource management that recognizes the complexity the chapter draws on Watson's (2010) pragmatic realism (Watson, 2010; Pihlström, 2023) and the theory of sociological imagination (Wright Mills, 1959; Bratton and Gold, 2015). The chapter puts forward the Collaborative Model framework (see Figure 1), which entails a more integrated tandem academia-practitioner that can engender informed practices in international human resource management, capitalizing on sensitive scholarship. The contribution of the chapter centers on its conceptualization of and emphasis on the recognition of multiple realities as a prerequisite for positive international human resource management research in an increasingly globalizing world.

## Re-imagining international HRM research methodologies

###  Social embeddedness of business research

The concept of phronesis is important here and is central to the study as the “moral compass” of the methodology, the objective is to critique the moral basis of management practice, which is largely contextual, given the multiple (and sometimes irreconcilable) interests. Phronesis integrates practical and moral knowledge (Githens, 2007). This would not overcome the substantive critique (that local methods struggle to deal with complex and contradictory social structures), but it could encourage researchers to hold a mirror to managerial practice in such a way as to engender critical reflection (even if many HR managers remain powerless in changing the structural conditions that affect their practice).

Habermas (1979) attaches great importance to the concept of “phronesis.” This is a significant development because knowledge is no longer local, but the effective HRM practitioner draws on strategic information from distant locations and global affairs. Habermas (1979) advocates the development of inquiry that does not neglect the practical daily concerns but integrates those practical concerns with larger moral, societal, and cultural issues. Additionally, he rejects the linear notion that theory should drive practice. Instead, he advocates the integration of theory into the daily lives of practitioners (Githens, 2007). This concept, while taking a pragmatist standpoint, divulges from the traditional modes of inference—induction and deduction—and complements it by a third mode—abduction—which is qualitatively different from the other two and utilizes methods from both (Svennevig, 2001); apparently, to bridge this discovery-design divide. In fact, the concept of abduction is originally attributed to Aristotle (Svennevig, 2001; Massingham, 2019). However, Cascio (2015) contends that it is not an easy task to conduct research that is useful, both for theory and for practice, as there is no one best way to do it. Yet, the field of IHRM is a broad field that can accommodate a variety of approaches to generating knowledge that is useful both to academics and practitioners (Cascio, 2015).

###  Sociological imagination

In terms of the sociological imagination approach, it is essential to examine the linkages with engaged research with regard to the development of a model of academia-practice partnership. Engaged research entails that the researcher meaningfully connects with various stakeholders at all stages of the process of inquiry (Bratton and Gold, 2015). This runs through the problem formulation, data finding, evaluation, and dissemination stages. This partnership, thus, entails that researchers and stakeholders are co-producers of knowledge; it is important to reiterate that, in the context of international HRM, stakeholders transcend the locality and the realms of the nation-state. This is an aspect of thought that is central to Wright-Mills's approach in “The Sociological Imagination,” where the author articulates an integral, engaged notion of “public sociology.” Sociology, as the study of society and culture, represents a critical framework for looking at organizational facts that transcend the locality (Flood and Fennell, 1995; Lanuza, 2011; Grothe-Hammer and Kohl, 2020). A pragmatist can strive to develop knowledge that can more realistically be the foundation of an actual or potential action than what has come before. The guide-to-action piece of HRM research proves to be more helpful to those who read it—whoever they may be and whatever the projects they are undertaking.

While equating Mills' “Sociological Imagination” (SI) (Mills, 1959) with what he called “social scientific imagination,” Watson (2010, p. 927) argues for working in a primarily “social scientific manner” which does not mean abandoning an aspiration toward critique but adopting a position which sees social science as a critical enterprise by its very nature. Watson furthermore states that SI relates to a context of global competitive pressures wherein all taken-for-granted assumptions about human resource management practices would be treated skeptically (Watson, 2007, 2010, and it is in this spirit that a pragmatic-realist critical-analytical study of human resource management would work. He suggests that the realities of HRM need to be seen in a global economic, political, and socio-cultural context (Watson, 2010). Sociological imagination, more than ever before, is needed in a global economy that transcends traditional rules and is dominated by unpredictability. This theoretical perspective is very relevant to international human resource management, whose issues are less predictable than those of domestic HRM.

###  Strengthening theorization through pragmatic integration

Scholars are increasingly advocating that research ought to complete a virtuous circle of theory and practice whereby research on managerial practice informs practically derived theory, which in turn informs managerial practice. However, the theory can inform practice effectively if it is an inclusive theory, i.e., which draws on the diversity of organizational contexts that are culture-bound. This duality theory-practice contributes to enhancing relevant and practical management knowledge (Rynes et al., 2001; Starkey and Madan, 2001; Van de Ven and Johnson, 2006; Chung et al., 2012), but it can be effective only if it is meaningful to the diversity of actors and professional involved in the management of human resources internationally. The academics further state that HRM research needs to be an engaged research, i.e., to engage with both the world of theory and practice; the questions addressed, in particular, should entail this interaction between two worlds rather than either on their own (Beer, 2015; Beer et al., 2015; Cascio, 2015). From a contextual and sociological standpoint, Beer et al. (2015) suggest that HRM research has so far neglected the distanced geographical locations, thus encouraging the case for IHRM. They state that the financial measures of HRM have been a particularly powerful factor in the Anglo-Saxon liberal market, which has been at the center of most of the work on HRM. In these countries, the study of HRM has tended to concentrate on private-sector organizations, particularly the more researchable and supportive prominent multinational corporations (MNCs). In terms of IHRM, they contend that it is the most localized management practice, and except for the top executives of MNCs, there is little “convergence” in the way HRM is conceived and managed and what effects its specific practices have in different geographic locations. In fact, geographic location particularly affects how HRM is understood, which stakeholders it is meant to serve, and what (HRM) practices have legitimacy (Beer et al., 2015). Additionally, Watson contends that HRM research requires bringing together, on the one hand, those who investigate how employment management practices might enhance the “performance” or “competitive advantage” and actors who currently dissociate themselves from such work by attaching the word “critical” to their work. For example, Hack-Polay (2012), studying homesickness among expatriates, found that it is a health problem that impacts performance and talent pool. He advocates that solutions are embedded in practical strategies, e.g., pre-departure training, collaboration between host and expatriates, etc.

In this perspective, Watson (2010) argues that critical research in human resource management is strengthened by the sociological imagination. Boxall (2021) believe that such an approach is part of the building block of effective theory. There is, therefore, strong support for the activation of a new paradigm in human resource management research. This would be more pragmatic and reflect better the way in which managerial action takes place in organizational settings, in time, and in space.

## Pragmatism as a dialogic approach to research in IHRM

We looked at the traditional interpretivist paradigm to see whether this study fits within its characteristics. Interpretivism is researcher-led through a sense-making process. This entails that the researcher attaches meaning to the participants' actions and narratives. Interpretivism is viewed as part of a process of theorization, legitimately based on drawing meaning from data. This research seeks to generate data whose intelligibility (that is, in terms of meaning) will predominantly depend on the interlocutors' own assessment of their meaning and not something imposed from the researcher's perspective. This is not to criticize the epistemological validity of interpretivism but to assert that other possibilities and paradigms are open. Within pragmatic HRM research, investigators ought to play the role of dialogue facilitators between the participants of all backgrounds, taking into account racial, cultural, gender, and religious divides, and the audience owing to the necessity to bring out participants' real narratives and experiences of organizations.

Major research questions ought to focus on “how the research participants explain to their audience the reasons for the way they feel about management; what core message they wish to convey to their interlocutors; how they would enact change to improve human resource practices in a given context.” Such questions have the potency to create a sense of interactive dialogue capable of generating additional questions and responses between the researchers and the participants. It should also be emphasized that it is important to translate indigenous words and understand how HRM is viewed and applied in different cultural contexts to fully comprehend the pragmatism being discussed. The collaborative model is schematized in the Figure 1.

As shown in Figure 1, effective human resource practice, in times of crisis in particular, can be significantly multidimensional and depend on a cultural dialogue between communities and wider audiences. This dialogue can be facilitated by the researcher who asks the right questions, which helps develop the right interpretation through consistent sense-making, consistent with the interpretivist paradigm. Effectiveness in human resource practice is also enacted by a collective conversation with their customers, bankers, and the wider community; this conversation or dialogue is as we discussed above. It can be included in research on the fundamental reasons behind the success or failure of organizational and people management policies and frameworks. Such conversational research into family businesses is more of an empirical nature that allows meaning and sense-making to be performed by readers and participants alike as they both seek to make sense of the complexities behind issues of social inclusion and exclusion, management success or failure, cultural acceptance or rejection, and organizational research.

## Pedagogical implications

This chapter has significant implications for research methods pedagogy. The understanding of the link between HRM practice and theory requires emphasis when teaching research methods to students in our universities. Students are co-producers of knowledge, and as future investigators, they need to learn to position themselves as social actors who are embedded in a cultural context and are entitled to appreciate their role as researchers and participants in that social system.

Teaching HRM research methods should, therefore, encapsulate a degree of culture teaching and the dynamics of globalization to delineate it from the heavy emphasis on the Western context and assure modern objectivity (Hack-Polay, 2018). This approach to teaching research methods will show that research or theory is not divorced from its societal, cultural, and institutional context. In the same way, because researching international HRM cannot be divorced from its socio-cultural and institutional context, the teaching of research methods in IHRM cannot. Students need to be introduced to culture, economics, and globality, which makes research make sense, particularly to Generation Z and Generation I, whose social and professional realities now transcend the locality.

Pragmatism in HRM research and teaching ought to be engaged research that is valuable for science and society. This contribution does not downplay the value of theoretical research but, on the contrary, brings value and elevates it to a different dimension. This, too, can inform practice.

## Conclusion

This chapter has introduced the perspective that theorization in human resource management needs to go through research that is made meaningful in the collaboration model. From this standpoint, finding solutions to organizations' problems is viewed as deeply rooted in the broader societal environment. Such a perspective supports a necessary integration of theory and practice. For example, Hack-Polay (2020), studying homesickness among expatriates, found that it is a health problem that impacts performance and talent pool. He advocates that solutions are embedded in practical strategies, e.g., pre-departure training, collaboration between host and expatriates, etc. This chapter is a reminder that a constructive HRM research approach must not be divorced from the institutional and social contexts, thus pragmatism (Boxall, 2021). This is what we term a collaborative research approach in human resource management research.

To achieve a higher degree of harmonization of rhetoric and deed, HRM must use collaborative qualitative that can engender intelligible and useful theories. HRM research cannot also underplay a positivist perspective, which can test existing HRM theories (Beer, 2015). The author contends that without these methods, the HRM field will stay narrow and will fail to provide useful or actionable knowledge; the field must reorient itself to produce useful and practical knowledge to stay relevant for practice. This gap between theory and practice, to the authors, appears artificial and stands in the way of answering effectively vital organizational and societal questions. Relevant praxis in creating IHRM value chains that can be creditably implemented does “require a close connection between human resource theory and practice beyond some heavily theoretical modern human resource paradigms” (Beer et al., 2015, p. 434). This is where the advocated collaborative model (academia-practice), in this study, can be a framework that helps make sense of reality for turning ideas into praxis and using praxis for further idea (theory) generation. In this respect, Mills' notion of the Sociological Imagination provides us with the perspective for studying IHRM in a critical style and for giving attention to the relationship between the personal circumstances of individuals in work organizations and the public issues that are raised by current and emerging employment management practices (Watson, 2010). This supports our view that phronesis has a central place in pragmatism.

## Data availability statement

The original contributions presented in the study are included in the article/supplementary material, further inquiries can be directed to the corresponding author.

## Author contributions

DH-P: Conceptualization, Resources, Writing – original draft, Writing – review & editing.
